# iPSC-Derived Astrocytes and Neurons Replicate Brain Gene Expression, Epigenetic, Cell Morphology and Connectivity Alterations Found in Autism

**DOI:** 10.3390/cells13131095

**Published:** 2024-06-25

**Authors:** Hamid Mostafavi Abdolmaleky, Reza Alam, Shabnam Nohesara, Richard C. Deth, Jin-Rong Zhou

**Affiliations:** 1 Nutrition/Metabolism Laboratory, Beth Israel Deaconess Medical Center, Harvard Medical School, Boston, MA 02215, USA; rezaalammba@gmail.com; 2 Department of Medicine (Biomedical Genetics), Boston University Chobanian & Avedisian School of Medicine, Boston, MA 02118, USA; snohesar@bu.edu; 3 Department of Pharmaceutical Sciences, Nova Southeastern University, Fort Lauderdale, FL 33328, USA; rdeth@nova.edu

**Keywords:** autism, epigenetic, gene expression, iPSC, astrocyte, neuron, brain

## Abstract

Excessive inflammatory reactions and oxidative stress are well-recognized molecular findings in autism and these processes can affect or be affected by the epigenetic landscape. Nonetheless, adequate therapeutics are unavailable, as patient-specific brain molecular markers for individualized therapies remain challenging. Methods: We used iPSC-derived neurons and astrocytes of patients with autism vs. controls (5/group) to examine whether they replicate the postmortem brain expression/epigenetic alterations of autism. Additionally, DNA methylation of 10 postmortem brain samples (5/group) was analyzed for genes affected in PSC-derived cells. Results: We found hyperexpression of *TGFB1*, *TGFB2*, *IL6* and *IFI16* and decreased expression of *HAP1*, *SIRT1*, *NURR1*, *RELN*, *GPX1*, *EN2*, *SLC1A2* and *SLC1A3* in the astrocytes of patients with autism, along with DNA hypomethylation of *TGFB2*, *IL6*, *TNFA* and *EN2* gene promoters and a decrease in *HAP1* promoter 5-hydroxymethylation in the astrocytes of patients with autism. In neurons, *HAP1* and *IL6* expression trended alike. While *HAP1* promoter was hypermethylated in neurons, *IFI16* and *SLC1A3* promoters were hypomethylated and *TGFB2* exhibited increased promoter 5-hydroxymethlation. We also found a reduction in neuronal arborization, spine size, growth rate, and migration, but increased astrocyte size and a reduced growth rate in autism. In postmortem brain samples, we found DNA hypomethylation of *TGFB2* and *IFI16* promoter regions, but DNA hypermethylation of *HAP1* and *SLC1A2* promoters in autism. Conclusion: Autism-associated expression/epigenetic alterations in iPSC-derived cells replicated those reported in the literature, making them appropriate surrogates to study disease pathogenesis or patient-specific therapeutics.

## 1. Introduction

Autism is a major neuro-developmental disorder affecting children whose prevalence has steadily increased in the past several decades [1]. Genetic defects contribute only ~10% of the disease risk, and environmental/nutritional factors affecting the epigenetic landscape appear to play a significant role in its etiopathology. Despite largely unknown etiologic factors, excessive inflammatory reactions and accompanying vulnerability to oxidative stress are well-recognized molecular findings in postmortem brain studies of autism. In particular, brain inflammation is a hallmark of autism [2], which is generally associated with widespread neuronal loss in postmortem studies of the brain tissue of affected individuals.

Several lines of evidence indicate that the head size of patients with autism is larger than that of individuals without autism [3]. This is accompanied by increased neuron number in the prefrontal cortex and higher brain weight and increased brain volume [4,5], reduced synapse elimination [6] higher spine densities [7] and an increase in BDNF level [8]. However, despite these early life brain alterations that favor higher brain cell growth potential, there is also evidence that as patients go into later childhood periods, their brain weight, cortical thickness, and cognitive capabilities are decreased, accompanied by the loss of neuronal arborization and increased spine density, but with immature morphology [9,10].

In addition to these neuro-anatomical alterations, there are also reports on widespread neuro-inflammation and increased oxidative stress in different parts of the brain of patients with autism [11]. Research data also indicate decreases in the glutathione/oxidized glutathione ratio and redox antioxidant capacity, and the increased oxidative stress in the brain of patients with autism may have consequences such as chronic inflammation and increases in mitochondrial superoxide production, protein oxidation and DNA damage [12,13]. Inflammatory and immune dysfunction includes elevated brain-specific antibodies against neuron-axon filament protein (NAFP) and glial fibrillary acidic protein (GFAP) [14], as well as chronic microglial activation that may mediate the dysfunction of glutamatergic excitatory receptors [15], increases in the levels of TGFB1, TNFA, IL-6 and IL-17 and other inflammatory cytokines [16,17,18], and down-regulation of *IL-2* in the blood cells of patients with autism [19]. Interestingly, while TNFα, inhibits methionine synthase (*MTR*) expression in cultured neuronal cells, and *MTR* expression is very high in the postmortem brains of normal individuals in early developmental periods (but decreases as age increases), in patients with autism not only is its early life peak absent but also a life-long decrease in *MTR* expression is observed compared to the control subjects [20]. This suggests that the reduced expression of *MTR*, which is involved in the functionality of methylation machinery, might be due to inflammatory processes in autistic brains [20].

Although inflammation and associated oxidative stress and epigenetic alterations are considered key components of autism pathogenesis [21,22,23], evidence from transcriptome profiling suggests that the increased inflammatory processes in autism might be due to the lack of functionality of anti-inflammatory mechanisms. Particularly, *IFI16* (interferon, gamma-inducible protein 16), an anti-inflammatory and innate antiviral gene, is decreased in the blood cells of infants and toddlers at risk for autism [24], but it is increased in the plasma of patients with autism [25]. There is also a report of increased expression of *IFI16* in the brain of these patients [26]. *IFI16* is involved in the innate immune response by recognizing viral dsDNA in the cell nucleus, providing the very last defense component in DNA protection against intrusive viral elements penetrating the cell nucleus, and *IFI16* hypoactivity in blood cells may make the genome vulnerable to viral intrusion.

In addition to proinflammatory genes, there are also reports of reduced expression of several key neuronal genes such as *RELN*, *SLC1A2*, *GAD1*, *TSC1* and *HAP1* in autism or autism spectrum disorders [27,28,29,30,31], which have not been causally linked to neuro-inflammation in autism. Despite these interesting findings in postmortem brain samples or blood cells in autism, the study of the developing brain in humans and the potential influence of environmental or therapeutic factors in disease pathogenesis or prevention and treatment in children with autism is challenging. Taking into account the limitations in the availability of brain samples of affected individuals for molecular studies of the pathogenesis or therapeutic interventions, we used the novel approach of iPSC-derived neuronal stem cells (NSCs), neurons, and astrocytes of patients with autism vs. control subjects to identify whether these model cell lines represent similar molecular alterations to those reported in the brain tissues or blood cells of patients with autism and to learn about the underlying cell type-specific molecular mechanisms which are involved in autism pathogenesis. Considering recent findings in the postmortem brain and peripheral tissues of patients with autism, as described above, we focused primarily on gene expression and epigenetic alterations of inflammatory, anti-inflammatory, and other key affected genes involved in neuronal growth rate, arborization and synapse formation in the differentiated cells. Here we present data indicating that a large fraction of genes whose expression or DNA methylation is affected in the blood or postmortem brains of patients with autism are also affected in the iPSC-derived neurons and astrocytes of these patients.

## 2. Materials and Methods

### 2.1. Samples

Five pairs of induced stem cells (iPSCs) generated from patients with idiopathic autism and normal controls, plus one iPSC line from a patient with fragile X mental retardation, were obtained from the NIMH (one/group) and Simons Foundation (Table 1). The samples from the Simons Foundation include iPSCs SV0001455, SV0001464, SS0013111 and SV0000525 from the control subjects, and SV0001473, SS0013099 and SV0000540 from patients with autism. Samples from the NIMH include iPSCs SC173 (control), SC171 (autism) and SC126 (fragile X mental retardation with autistic symptoms).

In addition to iPSC lines, we used 10 DNA samples extracted from the postmortem brain samples of five patients with autism (four male, one female) and five control subjects (four male, one female) for DNA methylation analysis. These 10 samples are a part of the samples previously obtained from the Autism Tissue Program, now part of the Autism Brain Network (http://www.autismbrainnet.org, and the Harvard Brain Tissue Resource Center (http://brainbank.mclean.org). To have more homogeneous samples, we only used DNA samples extracted from the frontal cortex of cases and controls in the study. The average age of the cases and control subjects were almost the same (8 years, vs. 8.6; SD, 1.73 vs. 2.88). The average postmortem interval was also the same (8.2 h in both groups; SD 3.56 vs. 3.42). For the diagnosis of all samples, we relied on the information collected by the biorepositories.

### 2.2. iPSC Maintenance and Cell Expansion

iPSCs were differentiated into NSCs, neurons and astrocytic cell types using Thermofisher standard protocols (https://www.thermofisher.com/us/en/home/references/protocols/neurobiology/neurobiology-protocols/differentiating-neural-stem-cells-into-neurons-and-glial-cells.html, accessed on 11 April 2024). In brief, human iPSC lines of normal controls and patients with autism were cultured in a 2D cell culture system in StemFlex™ Basal Medium supplemented with 10% StemFlex™ Supplement 10X (Thermo Fisher, Waltham, MA, USA, catalog #A3349401) 1% antibiotic (Antibiotic-Antimycotic, Thermo Fisher, Waltham, MA, USA, Cat# 15240062), 1% GlutaMAX-I Supplement (Thermo Fisher, Cat# 35050061) and 5 µL/mL of RevitaCell™ Supplement (Thermo Fisher, Cat#A2644501) on dishes incubated for 2 h at room temperature with Geltrex (Geltrex™ hESC-Qualified, Ready-To-Use, Reduced Growth Factor Basement Membrane Matrix Thermo Fisher Cat# 12760). Cells then were incubated at 37 °C (5% CO_2_) for 24 h, the medium was changed without the addition of the RevitaCell™ Supplement. After reaching a confluency of approximately 70%, cells were dissociated from wells using StemPro™ Accutase™ Cell Dissociation Reagent (Thermo Fisher Cat# A1110501), diluted 6-fold with PBS, centrifuged for 4 min (800 RPM at room temperature) and passed to new wells as described above.

### 2.3. Differentiation of iPSCs to Neuronal Stem Cells (NSC), Astrocytes and Neurons

After two passages of iPSCs lines, cells were differentiated into NSCs using Neuronal Induction Medium (Thermo Fisher, Cat# A1647801) consisting of KnockOut D-MEM/F12 medium supplemented with 1% STEMpro neuronal supplement (Thermo Fisher Cat # A1050801), FGF-basic (AA 10-155), Recombinant Human (bFGF) (Thermo Fisher Cat # PHG0024), EGF recombinant human (Thermo Fisher Cat# PHG0014), plus 1% GlutaMAX-I, antibiotic, and 5 µL/mL RevitaCell™ Supplement on dishes covered by Geltrex as mentioned above. After 3 passages, NSCs were cultured in SCIVAX 3D cell culture system (InfiniteBio, Sunnyvale, CA, USA, Cat# NCP-LSH96-2) following 3 days of culture in V-bottom shape wells to investigate the pattern of their growth states.

In the 2D culture system, similarly, after three passages, NSCs were allowed to differentiate into neurons by culturing on dishes already incubated with 20 μg/mL poly-L-Ornithine (MilliporeSigma, Burlington, MA, USA, Cat# P3655) for 1 h at 37 °C (2 mL for 35 mm dish). The culture vessels were rinsed twice with sterile water, followed by incubation with 10 μg/mL laminin (Thermo Fisher Cat# 23017) for 2 h at 37 °C and covered with PBS without calcium or magnesium until use.

For neuronal differentiation, we used a neurobasal medium and B-27 supplement, plus an antibiotic and 1% cultureOne supplement (Thermo Fisher Cat# A3320201). For astrocyte differentiation, NSCs were cultured in dishes incubated with Geltrex, and after two days, the culture medium was changed to astrocyte differentiation medium consisting of DMEM, 1% FBS, 1% Gibco N2 Supplement (Thermo Fisher Cat# 17502), 1% GlutaMAX-I and antibiotic. Note that all the media were prepared for 1 month and kept dark at 4 °C; only the required amount for that day was warmed up to 37 °C. Additionally, only for the first round of iPSC or NSC culture, was the RevitaCell™ Supplement added to the culture medium.

### 2.4. Immunocytochemistry

After three weeks of cell differentiation, the acquisition of neurons and astrocytes was confirmed with immunohistochemical staining with 1/200 diluted primary antibodies (e.g., Mouse IgG1 MAP2, Invitrogen, Cat # MA5--12826 for neurons and Rabbit GFAP or Mouse IgM A2B5, Invitrogen, Waltham, MA, USA, Cat # MA1-90445 for astrocytes) and corresponding secondary antibodies. In brief, cells were washed with PBS once after removing the medium and incubated with 4% paraformaldehyde at room temperature for 15 min. Cells were then, rinsed 3 times with D-PBS containing Ca^2+^ and Mg^2+^ and incubated 30 min in blocking buffer (1% BSA, 0.1% Triton™-X and 5% serum of the secondary antibody host species in D-PBS with Ca^2+^ and Mg^2+^). Next, after removing the blocking buffer, cells were exposed to primary antibody diluted in 5% serum and incubated at 4 °C overnight. The next day, cells were washed for 5 min with D-PBS containing Ca^2+^ and Mg^2+^ 3 times and were exposed to 1/1000 fluorescence-labeled secondary antibody (e.g., Alexa Fluor 594 goat anti-mouse IgM, Invitrogen, Cat# 1044, red color; Alexa Fluor 488 goat anti-mouse IgG, Invitrogen, Cat# A21029, green color; Alexa Fluor 350 goat anti-rabbit, IgG, Invitrogen, Cat# A21068, blue color, in 5% serum in PBS with Ca^2+^ and Mg^2+^) and incubated at 37 °C for 45 min in the dark, then washed 3 times with PBS and counter-stained with DAPI solution (Thermo Scientific, Waltham, MA, USA, Cat# 62248, 3 ng/mL, for 5 min), rinsed with D-PBS and covered with mounting medium (VectaMount, Cat# NC9611543, Fisher Scientific, Waltham, MA, USA). Astrocyte shape and phenotype were quite different from neurons, and their identity was confirmed by expression analysis of well-known astrocytic markers (e.g., A2B5 or GFAP).

### 2.5. DNA and RNA Extraction and cDNA Synthesis

Triplicate cultures of differentiated cells from each case and control subject were harvested and combined for DNA and RNA extraction using TRIzol (ZYMO Research, Direct-zol DNA/RNA MiniPrep, Irvine, CA, USA, Cat# R2080) according to the manufacturer’s instructions. The extracted RNA and DNA quality were checked using NanoDrop One (Thermo Scientific). One µg of total RNA was used for subsequent cDNA synthesis using SuperScript IV Reverse transcriptase (Invitrogen Life Technologies, Waltham, MA, USA, Cat# 18090050).

### 2.6. Gene Expression Analysis

Following cDNA synthesis, SYBR Green master mix (Applied biosystem, Carlsbad, CA, USA, Cat# A25742) and gene-specific primers (Supplementary Table S1) were used to perform qRT-PCR analysis (Biorad, CFX384 Touch™ Real-Time PCR Detection System, Hercules, CA, USA) normalized to β-actin gene expression using the standard ΔΔC_T_ method, following cycle threshold (C_T_) processing by the CFX software (version 3.1). Additionally, SRY gene expression analysis was used to determine or confirm the sex of all samples. Note that, in a correlation analysis of β-actin and GAPDH gene expression levels in NSCs and differentiated cells of cases and controls we found that the expression of these two genes is highly correlated (r = 0.94). Therefore, β-actin expression was used for the normalization of gene expression levels. Melting curves of PCR products were examined to verify that primer pairs generate a single product. Before real-time PCR, the PCR products from all primer pairs were analyzed using acrylamide gel electrophoresis to ensure that the primer pairs produced a single product of the expected size.

### 2.7. DNA Methylation Analysis

For DNA methylation analysis, enzymatic digestion of extracted DNA coupled with the qPCR method was used to evaluate the levels of 5-mC (5-methylcytosine) as well as 5-hmC (5-hydroxymethylcytosine) in the gene promoter region [32]. To differentiate between 5-mc and 5-hmc, we used qPCR analysis of MspI and HpaII restricted DNA treated by T4 Phage β-glucosyltransferase (T4-BGT) + uridine diphosphoglucose (UDP-Glu) using the EpiMark 5-hmC and 5-mC Analysis Kit, (https://www.neb.com/en-us/products/e3317-epimark-5-hmc-and-5-mc-analysis-kit, accessed on 11 April 2024) according to the manufacturer’s instructions (New England BioLabs, Ipswich, MA, USA, Cat# E3317S) where MspI and HpaII unrestricted DNA represent 5-hmC and 5-mC, respectively. This was the primary method for epigenetic analysis of candidate genes using primers flanking CCGG sequences (Supplementary Table S2). As stated previously, in addition to acrylamide gel electrophoresis, the melting curves of PCR products were examined to confirm that primer pairs produce a single product. For the quantification of 5-mC and 5-hmC levels, the ΔΔC_T_ method was used, normalized with the C_T_ of uncut DNA of the target gene’s promoter regions, and a two-tailed *t-*test was used for data analysis.

### 2.8. Evaluation of Growth Rate in NSCs and Differentiated Cells and Neuronal Migration Assay

The same numbers of NSCs were cultured in 96-well plates in triplicates, and after four days, their growth rates were evaluated using MTS and a Synergi H1 microplate reader (BioTek Instruments, Winooski, VT, USA). Additionally, the number of cells in each well was counted using a hemacytometer (Fisher Scientific, Cat# 0267151B) to confirm MTS results. We also estimated cell numbers by qPCR amplification of DNA obtained from each well using a SingleShot solution, with the elimination of DNase use indicated in the protocol (Biorad, Cat# 172-5080). In brief, 49 µL of Cell Lysis buffer and 1 µL of proteinase K solution were added to each well of 96-well plates containing cells already washed with PBS. After 10 min, cell lysate was transferred to PCR tubes and incubated at 37 °C for 5 min followed by 5 min at 75 °C. One µL of this solution was used for qPCR analysis using primers designed from the promoter region of multiple genes such as *TGFB2, RELN* and *HAP1*. Since each cell contains two chromosomes and two copies of DNA, the quantity of cells reflects the amount of DNA and thus cycle threshold (Ct) values in qPCR analysis. The ΔΔC_T_ method was used to estimate the relative quantity of DNA amplified in autism, normalized with the C_T_ of the amplified DNA from the control subjects. For example, if the average C_T_ of the control subjects and autism were 24 and 25, respectively, the amount of DNA (i.e., cells) was considered half in autism vs. controls. As primers were designed from the gene’s promoter regions, RNA was not amplified as a noise product during DNA amplification. We performed this analysis for multiple genes, including *TGFB2, HAP1* and *RELN*, to ensure that the promoter of the target gene(s) is not potentially in the coding region of another gene or affected by copy number variations. High levels of correlation (r = 0.9) of the PCR products of multiple genes indicated that the promoter regions of the target genes are not a part of the coding region of other genes or influenced by potential copy number variations. The same number of cells were cultured for astrocyte or neuronal differentiation, and after 3–4 weeks of differentiation, real-time PCR amplification of extracted DNA, as well as MTS (only for NSCs and astrocytes) and cell counting methods were used to assess cell growth rates. Notably, due to numerous dissimilar neuronal branches unevenly occupying the surface of culture dishes, the MTS method was deemed inappropriate for quantification.

To evaluate neuronal migration with minimal damage to long neuronal processes, which may impact cell viability, the same numbers of neurons were cultured in 12-well plates containing a coverslip in each well. After one week and almost 50% confluence, each coverslip was detached from the surface using a hook-shaped needle and a surgical mosquito to transfer the coverslip to another well already incubated with L-ornithine/laminin. The number of cells passing the border of the coverslip was counted every other day. Additionally, after one week, the coverslips were removed from the wells, and the remaining cells in the well (migrated cells) were counted using trypan blue. The amount of DNA in each well was also measured using qPCR as described above. Note that during cell culture experiments, we realized that the size and shape of differentiated cells are different in controls vs. those in autism. Therefore, we measured the size of 12 randomly selected iPSC-derived NSCs and astrocytes in triplicate for cases and controls. For these measurements, we utilized the formula of (length + width) divided by two. Given the difficulty in quantifying the size of neurons due to their complex arborizations, using an analytical pipeline, we assessed the number of neuronal arborizations in individual neurons, dendritic spine density per cm of neuron images and spine dimensions/size (mm), as measured on the ZOE Fluorescence Cell Imager screen (in which each mm represents ~2 µm) in cases compared to controls. We counted individual neurons for neuronal connectivity after two weeks of neuronal differentiation in culture. To estimate the number of single neurons, 12 fields of each well were surveyed for single neurons or neurons with less than two connections. For size quantification, we utilized the highest magnification of the ZOE Fluorescent Cell Imager, a digital microscope with a standard magnification of 175× that can zoom up to 700×. We also used the Neurite Outgrowth Staining Kit (Thermofisher, Cat# A15001) to evaluate general neuronal arborizations in the sample cases and controls according to the manufacturer’s instructions. For this experiment, 400 NSCs were seeded in each well of a 96-well plate, subjected to neuronal differentiation conditions, and after three weeks, their neuronal arborizations were quantified using a Synergy H1 microplate reader (BioTek).

### 2.9. Statistical Analysis

A two-tailed *t*-test was used for data analysis unless stated otherwise. An alpha value of less than 0.05 was considered significant. GraphPad Prism 10.1.2 software was used for data analysis, standard deviation calculation and figure generation. Notably, the cell shape and growth pattern of cells from the patient with Fragile X syndrome appeared to align more closely with those of normal controls. As a result, given that this particular sample was from a single line, while the other samples were obtained from patients with idiopathic autism, it was subsequently excluded from further statistical analyses.

## 3. Results

### 3.1. Verifying the Identity of Differentiated Cells Using Immunohistochemistry

**Figure 1:**
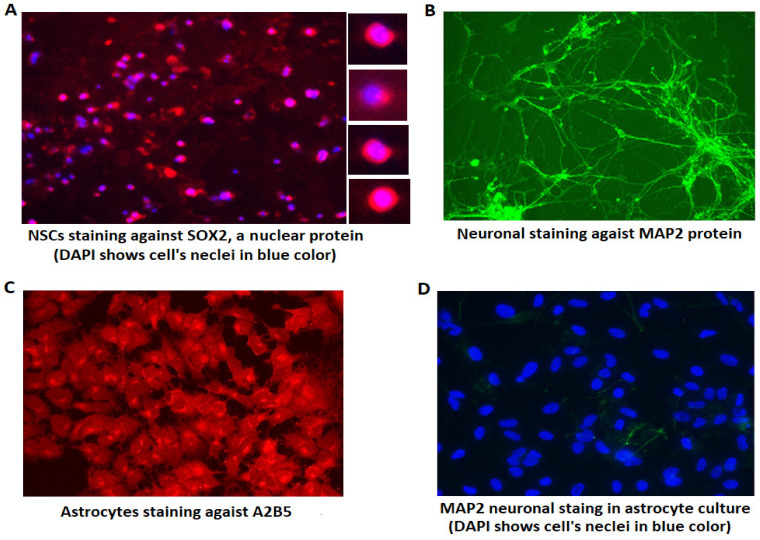
Immunocytochemistry analysis using different antibodies against cell marker proteins to verify the identity of differentiated cells. (**A**) NSCs are stained with a red fluorescent secondary antibody against an anti-SOX2 antibody. SOX2 is a nuclear protein. Four nuclei are shown with a larger magnification (700× vs. 175×) at the right side of the panel to illustrate the nuclear localization of SOX2. DAPI (blue) was used to label nuclei. (**B**) Neurons (175× magnification) are stained green against MAP2. (**C**) Astrocytes are stained with a red fluorescent secondary antibody against A2B5. (**D**) Astrocytes in culture were stained for MAP2. Since the red color of astrocytes obscures the green neuronal staining in (**D**), which is designed to detect potential neuronal impurity in astrocyte culture, only DAPI was used to label all cell nuclei, indicating the total cell number (75 nuclei in this field, with potentially two neurites stained in green). The microscope magnification in (**C**,**D**) is 350×.

Following iPSC differentiation into NSCs and neurons or astrocytes, an immunocytochemistry assay was used to confirm the identity of differentiated cells. As shown in Figure 1A, the identity of NSCs was confirmed using an antibody against SOX2, a cell nuclear protein. After 2 weeks of neuron and astrocyte differentiation, primary antibodies against MAP2 and A2B5 for neurons and astrocytes, respectively, along with the corresponding secondary antibodies, also confirmed the successful differentiation of neurons and astrocytes (Figure 1B,C). To investigate the specificity of cell differentiation, we stained differentiated astrocytes with MAP2 antibody and found only a few neurites among a large population of astrocytes in culture (i.e., two neurons out of 75 cells, Figure 1D), while the manufacturer of cell differentiation kit claims that the specificity of cell differentiation is almost 99%. An inverse experiment was conducted for neuronal cells (i.e., A2B5 staining for astrocytes against neurons in culture) with similar outcomes, indicating that the impurity of differentiated cells is trivial and negligible for subsequent studies. Additionally, to further confirm the efficacy of cell differentiation, we analyzed the expression level of *SOX2, MAP2* and *GFAP* (a marker of astrocytic cells) two weeks after NSC, neuron and astrocyte differentiation. While NSCs exhibited a large amount of *SOX2* expression, and there was no significant difference between cases and controls, its expression in neurons was <4% of NSCs (5 PCR cycles later than NSCs) and astrocytes had no *SOX2* expression. Moreover, *MAP2* expression in neurons was 110-fold higher than in astrocytes, and neuronal expression of *GFAP* was less than 2% of that in astrocytes.

### 3.2. Expression Alterations of NSCs and Differentiated Cells

Real-time PCR for the expression analysis of 25 genes (mostly inflammatory, proliferator, and genes related to cell connectivity as reviewed in the introduction) revealed increased expression of several inflammatory genes in cells of subjects with autism, particularly in astrocytes as shown in Table 2. *TGFB1* and *TGFB2* were among the most prominent genes, with approximately a 2-fold increased expression in autistic astrocytes and to a lesser extent in neurons. Notably, expression levels of *TGFB1* and *TGFB2* in astrocytes were 6- and 3-fold (respectively) higher than in neurons. To determine whether altered expression of *TGFB* genes occurred before or after differentiation, we analyzed *TGFB1, B2* and *TGFB3* gene expression in the NSCs of patients with autism and control subjects. Despite the variability of the gene expression levels in both groups, there were no significant expression alterations in the NSCs of patients with autism vs. controls, indicating that the aberrant expression pattern in autism started after differentiation. Remarkably, the expression levels of *TGFB1* and *TGFB3* were reduced in differentiated astrocytes and neurons vs. NSCs (almost half), but *TGFB2* exhibited a reverse trend (increased 2-fold in astrocytes and neurons vs. NSCs). Noteworthily, the expression of *NR2E1* (*TLX*), which regulates *TGFB2* expression [33] and is “a master regulator of neural stem cell maintenance and neurogenesis” [34], was not affected in differentiated cells.

*IL6*, an inflammatory gene with 30-fold greater expression in astrocytes vs. neurons, exhibited higher expression in the astrocytes and neurons (34-fold and 28-fold, respectively). However, *IL1B* exhibited a mild, but insignificant increase in these cell lines in autism vs. control subjects (Table 2). Furthermore, *TNFA*, a major inflammatory gene, showed no expression changes in autism compared to the control subjects. We also analyzed the expression of *TNFRSF1A*, a gene tightly linked to other inflammatory genes, and found no evidence for expression changes in NSCs, neurons and astrocytes in autism vs. control subjects (Table 2).

Regarding anti-inflammatory genes, while *IFI16* expression was significantly decreased in the NSCs of patients with autism (*p* = 0.005), its expression was increased in astrocytes (~50%, *p* = 0.02) and only insignificantly in the neurons of patients with autism. Notably, *IFI16* expression in astrocytes was 7-fold higher than in neurons. *IL4*, another anti-inflammatory gene with 3-fold higher expression in astrocytes vs. neurons and an overall low level of expression, exhibited 5-fold greater expression in astrocytes of controls vs. patients with autism (i.e., 80% decrease in autism, *p* = 0.04, Table 2).

On the other hand, *BDNF* expression was increased in the NSCs and astrocytes of patients with autism but not in neurons, and its expression in astrocytes was 5- and 3-fold higher than NSCs and neurons, respectively (Table 2). Interestingly, the expression of *NTRK2* (a receptor for BDNF), with almost 3-fold greater expression in astrocytes vs. neurons, exhibited a 30-fold decrease in the astrocytes of patients with autism vs. controls (*p* = 0.01), but not in neurons or NSCs. Similarly, *IGFBPL1*, with almost 2-fold greater expression in astrocytes vs. neurons, was a gene with >30-fold reduced expression in the astrocytes of patients with autism (*p* = 0.04) but was not significantly different in neurons (Table 2). Additionally, *RELN*, with almost no expression in NSCs and 2-fold greater expression levels in neurons vs. astrocytes, exhibited a highly significant 30-fold reduced expression in the astrocytes (but not in neurons) of patients with autism. Since TGFB1, mediated by snail (SNAI1), can inhibit *RELN* expression [35], we analyzed *SNAI1* expression in these cells and found no evidence for *SNAI1* expression alterations.

Engrailed Homeobox 2 gene (*EN2*) that, similar to *RELN*, controls pattern formation and dopaminergic neurogenesis during the development of the central nervous system and is linked to neuronal connectivity [36], trended for reduced expression in the NSCs and astrocytes of patients with autism, but in neurons exhibited increased expression (>3-fold, *p* = 0.05). Notably, *EN2* expression level in astrocytes was ~20-fold higher than in neurons.

*CXCR4*, a gene related to astrocyte survival [37] with 3-fold greater expression in astrocytes compared to neurons, showed decreased expression in the astrocytes (80%, *p* = 0.006) and to a lesser extent in the neurons (28%, *p* = 0.1) of patients with autism vs. controls (Table 2). *GPX1*, a gene that acts against oxidative stress [38], also exhibited decreased expression in the astrocytes of patients with autism (~40%, *p* = 0.015) but not quite a significant decrease in neurons (~30%, *p* = 0.05, one-tailed t-test) or in NSCs.

SLC1A2, a glucose/glutamate transporter, was among other genes whose expression was greatly reduced in the astrocytes (90%, *p* = 0.045) but insignificantly in the neurons (50%, *p* = 0.2) of patients with autism vs. controls. Like *TGFB2*, *SLC1A2* expression in astrocytes was higher than in neurons (2-fold). There was no significant alteration in *SLC1A2* expression in the NSCs of patients with autism vs. controls, suggesting that its expression alteration happens after astrocyte/neuronal differentiation. *SLC1A3*, which is similar to *SLC1A2*, involved in rapid glutamate removal from the synaptic cleft [28] and had >4-fold higher expression in astrocytes vs. neurons, was also decreased by 90% only in the astrocytes of patients with autism (*p* = 0.002, Table 2).

Additionally, *SIRT1* and *SNCA*, with the same levels of expression in astrocytes vs. neurons, and *NURR1* (with 2-fold expression in astrocytes vs. neurons), as well as *HAP1* exhibited highly significant decreases in expression in the astrocytes of autism, but not at statistically significant levels in neurons or NSC, except for *HAP1* which trended a decreased expression in neurons (Table 2). While *HAP1* expression in the controls’ neurons was almost 2-fold more than the controls’ astrocytes, its expression in autistic neurons and astrocytes was reduced by ~60% compared with the corresponding controls. Sinapsin-1 (*SYN1*), a gene specifically expressed in neurons, also exhibited a notable trend toward decreased expression in patients with autism, with a reduction of approximately 50% (*p* = 0.1, two-tailed *t*-test).

As the growth rate of NSCs was greater in patients with autism, we also analyzed the expression of several other genes that support cell proliferation, including *TWIST* (a transcription factor important in embryonic development), *IGF1* (an insulin-like growth factor) and Survivin (an apoptosis inhibitor) in NSCs which exhibited no changes in autism vs. controls. However, *MKI67*, a marker for cell proliferation, revealed a significantly increased expression in NSCs of patients with autism (*p* = 0.048).

### 3.3. Epigenetic Analysis of Affected Genes Regulated by DNA Methylation

**Figure 2:**
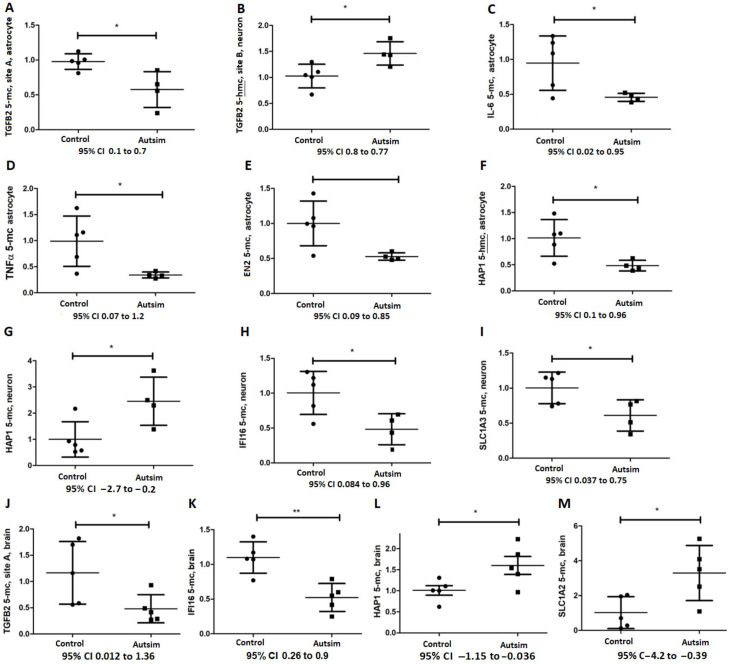
DNA methylation (5-mc) and hydroxy methylation (5-hmc) alterations in iPSC-derived astrocytes and neurons (**A**–**I**) as well as postmortem brain samples (**J**–**M**) of patients with autism vs. control subjects. The Y-axis represents Mean ± SEM for both the control group and the autism group. 95% confidence intervals (CI) are denoted for each gene under the X-axis. *p*-values between 0.05 and 0.01 are marked with *, and *p*-values less than 0.01 are marked with **.

Considering that many reports indicate epigenetic alterations in autism [18,39,40], we performed promoter DNA methylation analysis for several affected genes having CCGG sequences in their promoter region and known to be regulated by DNA methylation or exhibiting considerable expression alterations in the astrocytes or neurons of patients with autism. The *TGFB1* promoter region was almost totally unmethylated in iPSC-derived astrocytes in both the control and autism groups. However, neurons exhibited partial DNA methylation (nearly 50%, 5-mC) but no significant changes in cases vs. controls. Where almost half of all *TGFB1* promoter DNA methylation in neurons was due to 5-hmC, and the level of 5-hmC in autism was twice as much compared to the control subjects, this difference was not statistically significant. DNA methylation analyses of *TGFB2* revealed significant promoter DNA hypomethylation in the astrocytes of patients with autism at site A (Table 3 and Figure 2A). At site B (downstream of site A), while astrocytes exhibited totally unmethylated DNA, neurons were extensively methylated, highlighting an important distinction between neurons and astrocytes. Regarding 5-hmC, we found an increase in the 5-hmC level of *TGFB2* at site B in the neurons of patients with autism (~50%, Table 3 and Figure 2B). *IL6* and *TNFA* promoter regions were also hypomethylated in the astrocytes of patients with autism but not in the neurons (Table 3 and Figure 2C,D). Additionally, the promoter region of *EN2* exhibited reduced DNA methylation levels in the astrocytes of patients with autism (Table 3 and Figure 2E), though we found no evidence for 5-hmC alteration in autism vs. controls. However, we found a significant decrease in the 5-hmC level (i.e., relatively more 5-mC) of the *HAP1* promoter region flanking a progesterone binding site (CGCCCGCGC) in the astrocytes of patients with autism vs. controls (~50%, Table 3 and Figure 2F).

In neurons, we observed DNA hypermethylation (5-mc) of the same promoter region of *HAP1* in patients with autism (>200%, Table 3 and Figure 2G). Nonetheless, the level of 5-hmC was not different in the neurons of patients with autism vs. controls (Table 3). The promoter regions of *IFI16, SLC1A2*, and *SLC1A3* were largely unmethylated in astrocytes both in controls and patients with autism (Table 3) but were moderately methylated in neurons (signifying neuronal identity vs. astrocytes) where *IFI16* and *SLC1A3* were hypomethylated (~50%, and 40%, respectively, Table 3 and Figure 2H,I) and *SLC1A2* exhibited no change in autism (Table 3).

In postmortem brain tissues of five cases with autism and five control subjects, the *TGFB1* promoter region was extensively unmethylated (~5–10% methylation) and there was no significant change between cases and controls, but methylation in autism was 22% less than controls. Considering *TGFB2*, similar to iPSC-derived astrocytes, the target CpGs of site A exhibited DNA hypomethylation in the postmortem brain samples of patients with autism vs. controls (~50%, Mean ± SEM in controls and autism, 1.16 ± 0.27 and 0.4805 ± 0.1, respectively, *p* = 0.05, Figure 2J). Site B of *TGFB2* (which represents DNA methylation of neurons) was partially methylated, but there was no significant difference between cases and controls. As shown in Figure 2K,L, we also found that *IFI16* promoter DNA is hypomethylated (~40%, Mean ± SEM 1.1 ± 0.1 and 0.5 ± 0.1 in controls and autism, respectively, *p* = 0.003), but *HAP1* promoter was hypermethylated in the postmortem brains of these patients (~60%, Mean ± SEM 1 ± 0.11 and 1.6 ± 0.2 in controls and autism, respectively, *p* = 0.04). The *SLC1A2* promoter region was also hypermethylated (~300%, Mean ± SEM 1 ± 0.4 and 3.3 ± 0.7 in controls and autism, respectively, *p* = 0.02) in the brains of patients with autism (Figure 2M), though its overall promoter DNA methylation was trivial (~5% in controls and 15% in autism).

### 3.4. Alterations in Cells’ Growth Rate and Shape in Autism

**Figure 3:**
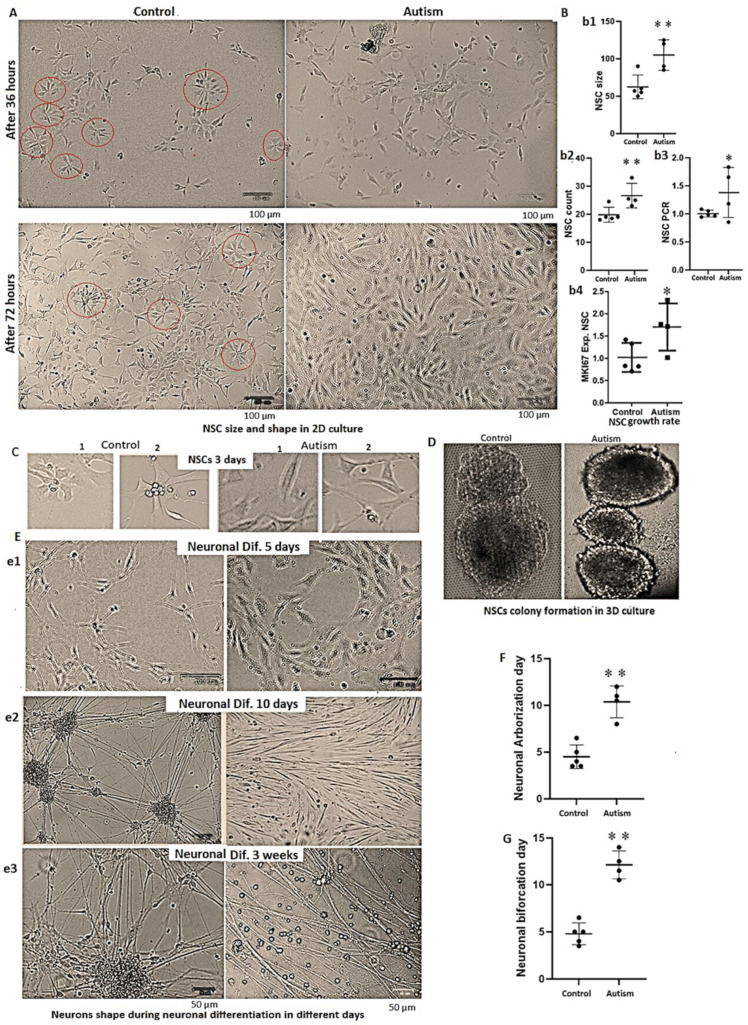
iPSC-derived NSC morphology and different features of neuron’s shape during neuronal differentiation in autism vs. controls. (**A**) iPSC-derived NSC colony shape and size for representative control and autism subjects after 36 and 72 h in culture. As demarcated with red circles, cell colonies in control subjects were predominantly star-shaped, but this morphology was rarely observed in cell colonies from individuals with autism (scale bars represent 100 µm). (**B**), b1. NSCs size is significantly larger in autism vs. control subjects (*p* = 0.003) as the size of 12 randomly selected cells per well (in triplicate for each case and control) was measured. (**B**), b2, b3 and b4. The growth rate of iPSC-derived NSCs from patients with autism is also increased compared to the control subjects using cell counting method (b2), or qPCR amplification of the extracted DNA normalized to the control subjects’ mean cycle threshold (Ct) value (b3), as described in Methods (triplicates were combined). The expression of MKI67, a marker of cell proliferation, was also increased in autism ((**B**), b4). (**C**) During differentiation in representative control subjects, the star-shaped pattern of NSCs and neurons and scrambled patterning in autism. (**D**) Connectivity of NSC colonies in controls vs. autism after 3 weeks of 3D cell culture. (**E**) Multi-angle cell patterning in control cells and needle or fusiform cell patterning in autistic cells after five days of neuronal differentiation (e1) and the following days (e2 and e3). (**F**,**G**) The delay in start days of arborization (**F**) and bifurcation (**G**) in autism vs. controls during neuronal differentiation in triplicates of cases and controls (*p* = 0.006 and *p* = 0.001, respectively). *p*-values between 0.05 and 0.01 are marked with *, and *p*-values less than 0.01 are marked with **.

We found that the size of NSCs from patients with autism was larger than control subjects (almost doubled, *p* = 0.0003, Figure 3A,B(b1)), and the NSCs from patients with autism had an elevated growth rate in the MTS analysis (*p* = 0.013, 1 in controls vs. 1.33 in autism, SD 0.084 vs. 0.125, respectively). Since the cell sizes were larger in autism, and the MTS assay was deemed improper as a growth assay, we employed the cell counting method and qPCR amplification of the extracted DNA (as described in Method), which confirmed an increased growth rate in NSCs from the patients with (>30%, *p* = 0.01) compared to control subjects (Figure 3B(b2,b3)). Additionally, *MKI67* expression, a marker for cell proliferation, revealed a significantly increased expression in autism (Figure 3B(b4)). Notably, in a 2D cell culture system the shape of most NSC colonies in normal controls was star-shaped as reported by others [41]. However, the shape of most NSC colonies from the idiopathic patients with autism was haphazard and somehow disorganized with a tilling pattern (Figure 3C), though this was not observed in the NSCs of a patient with fragile X syndrome. Our preliminary studies in a 3D culture system indicate that NSC colonies of controls exhibit a tendency for attachment and merging after 12 days, but colonies of the patients with autism remained detached for three weeks (examples shown in Figure 3D). However, this finding should be interpreted cautiously, as there were no other quantitative measures aside from the period remaining detached from other colonies in autism vs. controls.

### 3.5. Delay in Neuronal Differentiation and Reduced Neuronal Migration in Patients with Autism

**Figure 4:**
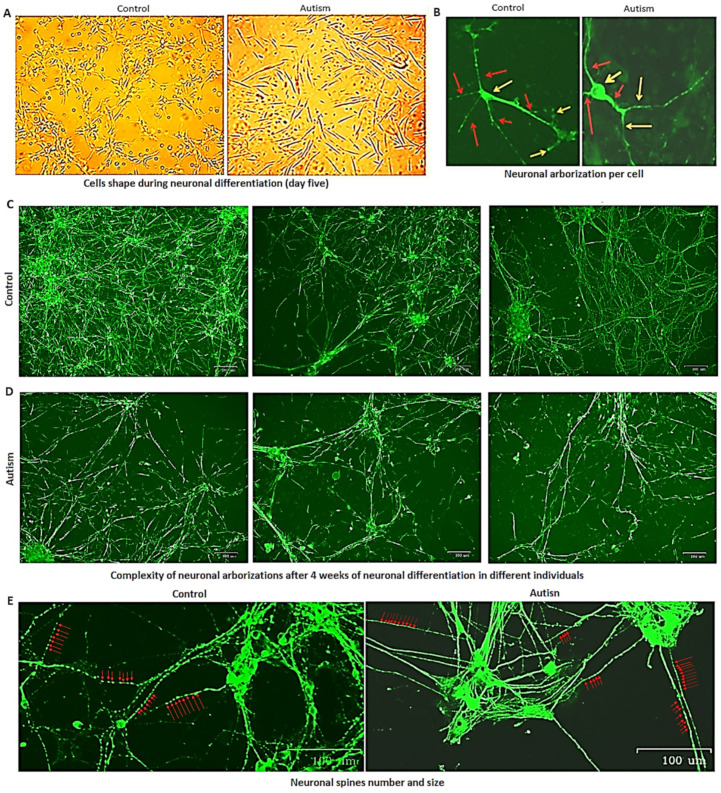
Neuronal network formation and spine density in autism vs. controls. (**A**) The shape of cells on day five of neuronal differentiation at 100× microscope magnification. (**B**) iPSC-derived neurons for a control subject and autism stained with MAP2 primary antibody and anti-mouse IgG. Representative neurons of a control subject and a patient with autism show five and three main processes, respectively (marked with red arrows). Some of these processes bifurcate to create more branches (marked with orange arrows) which are more frequent in control subjects. Microscope magnification is 700×, and cell nuclei are indicated with yellow arrows. (**C**,**D**) Comparison of neuronal networking in iPSC neurons of three controls (**C**) vs. three subjects with autism (**D**) exhibiting much more complex networking in the control subjects. The scale bars in (**C**,**D**) represent 100 µm. (**E**) Spines in autism are smaller in size, but their numbers are higher than controls, as marked with red arrows.

NSCs were differentiated into neurons as described in Methods. While the neuronal cells of controls after five days of differentiation were multi-angled and showed clear evidence of differentiation with visible neuronal arborization (Figure 3E, panel e1 and Figure 4A), autistic neurons were fusiform and showed minimal signs of neuronal differentiation for 10 days (Figure 3E, panel e2), and their neuronal processes exhibited limited connectivity for 3 weeks (Figure 3E, panel e3). While neuronal arborization and bifurcation were evident in the controls as early as day five of neuronal differentiation, in autism, they appeared almost five days later (*p* = 0.006 and 0.001, respectively, Figure 3F,G and Figure 4A). Neuronal connectivity was more complex and denser in the controls vs. patients with autism and the connection patterns of cells after differentiation into neurons were disorganized in patients with autism vs. controls (Figure 3E, panel e3).

Immunohistochemistry assays and the analytical pipeline also showed that neuronal arborization and bifurcation were reduced up to 50% in autism (*p* = 0.04, 6.3 vs. 3.4 in control and autism, respectively, Figure 4B), and network formation in the neuronal cells of patients with autism was less complex than controls along with reduced neuronal neuron-to-neuron connections in autism (Figure 4C,D). Neurite Outgrowth analysis revealed a significant reduction in neuronal arborization in the autism samples compared to the controls (32%, 1 vs. 0.68, *p* = 0.02). Notably, the network formation of the control neurons was predominantly pentagon-shaped (bees’ nest) with many collaterals, but in autism, it was mostly fusiform with end-to-end connections. Consistent with the findings of another study [7], we also found evidence for increased dendritic spine density in autism (~10%, 5.92 vs. 6.45 per cm on the ZOE Fluorescence Cell Imager screen, *p* = 0.044), but the spine size was nearly 20% smaller than controls (1.44 vs. 1.15 mm as measured on the ZOE Fluorescence Cell Imager screen, *p* = 0.046), Figure 4E.

Neuronal connectivity was also evaluated by counting single neurons (without any connection or with less than two connections) after two weeks of neuronal differentiation in culture. In the controls, a larger number of neurons exhibited multiple connections with neighboring neurons, but there was a significantly higher number of single neurons in autism (*p* = 0.016, 5.5 vs. 3.1, SD 1.4 vs. 0.8, in control and autism, respectively).

**Figure 5:**
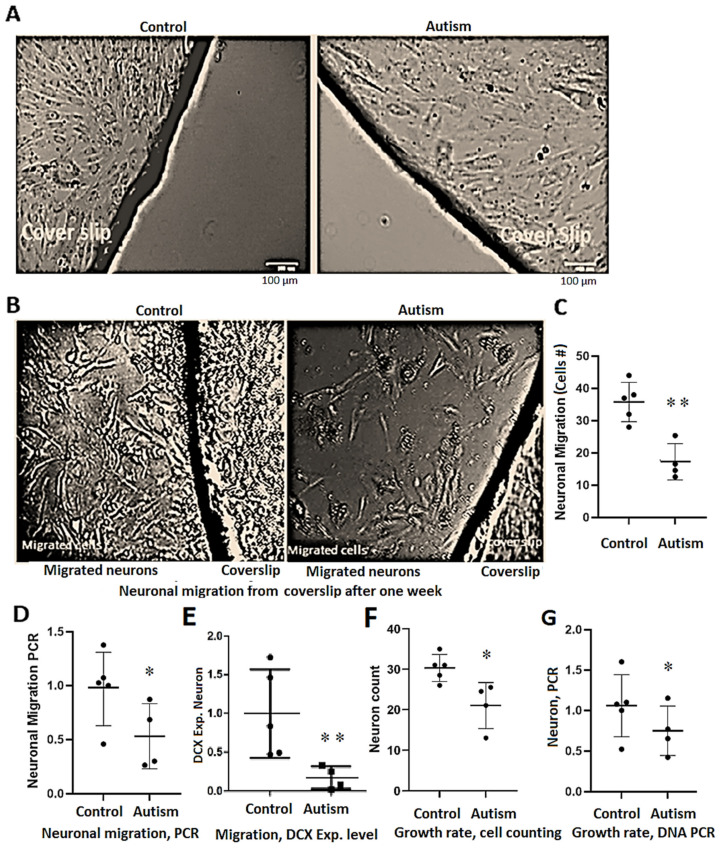
Neuronal migration and growth rate of neurons in controls vs. autism. (**A**) Coverslips (three for each case or control) containing neurons after 3–4 days of culture were transferred to new dishes, and cells migrating out of the border of the coverslips were counted every other day as well as after one week. (**A**) A representative sample of a control subject and a patient with autism on the first day (the scale bar represents 100 µm both for autism and the control subject). (**B**) The status of migration after one week. (**C**–**E**) Significant differences of migrated neurons in the control group vs. patients with autism (triplicate cultures were counted and averaged at day five of transferring the coverslips to new wells, based on cell counting method, DNA PCR and *DCX* expression level (a marker of neuronal migration), respectively. (**F**,**G**) Growth rate of iPSC-derived neurons from control vs. subjects with autism (in triplicate) based on cell counting method (**F**) or qPCR amplification of the extracted DNA normalized to the mean C_T_ value of the control subjects (**G**). As described in Methods for qPCR analysis, cells from the triplicate of each experiment were combined for DNA or RNA extraction for subsequent analyses. In panels (**D**,**G**), *p* values represent unilateral *t*-test results. *p*-values between 0.05 and 0.01 are marked with *, and *p*-values less than 0.01 are marked with **.

To evaluate neuronal migration in cases vs. controls without affecting neuronal structure/arborization during growth, neurons were cultured in 12-well plates containing 3 coverslips in each well. After almost 50% confluence, each coverslip was transferred to another well already coated with ornithine/laminin as described in Methods. A larger number of cells from control subjects passed the border of coverslips seven days after the relocation of the coverslip to the new wells (Figure 5A,B). Our daily observations and cell counting indicated that the number of migrated neurons for control subjects was >2-fold higher than that for patients with autism at day five (*p* = 0.004, Figure 5C). After one week, the coverslips were removed from the wells, and the remaining cells were counted as described in Methods. This quantitative approach also showed that the migrated neurons in control subjects are almost 3-fold more than in patients with autism (*p* = 0.002). qPCR amplification of the extracted DNA revealed that the average C_T_ of the control subjects is 1.2 cycles lower than the C_T_ of patients with autism indicating a 2.3-fold higher neuronal migration in the controls compared to the patients with autism (*p* = 0.04, one tail t-test, Figure 5D). Expression analysis for *DCX* transcript, a marker of neuronal migration, revealed 80% reduced expression in autism (Figure 5E), while *MKI67* expression level was the same in both groups.

### 3.6. Reduced Growth Rate and Long-Term Neuronal Survival in Autism vs. Controls

An equal number of NSCs were cultured and underwent neuronal differentiation after three days, and four weeks later, the number of neuronal cells was evaluated through cell counting methods (utilizing trypan blue) and real-time PCR amplification of their extracted DNA, as described methods. Even though the NSC growth rate in autism is greater than the control subjects, both methods revealed a 30% reduction in the growth rate of neurons in autism (*p* = 0.05, in two-tailed and one-tailed t-test analysis for cell counting and PCR amplification methods, respectively), as shown in Figure 5F,G. Regarding long-term neuronal survival, neurons of the control subjects could survive for >10 months upon multiple passages of the coverslips to new wells (two lines were investigated). However, their growth rate, arborization potential, and network formation decreased as cells aged. The decrease in growth rate and network formation was more prominent in patients with autism, and their long-term survival was compromised after four months).

### 3.7. Increased Astrocyte Size, Reduced Growth Rate and Long-Term Survival in Patients with Autism

**Figure 6:**
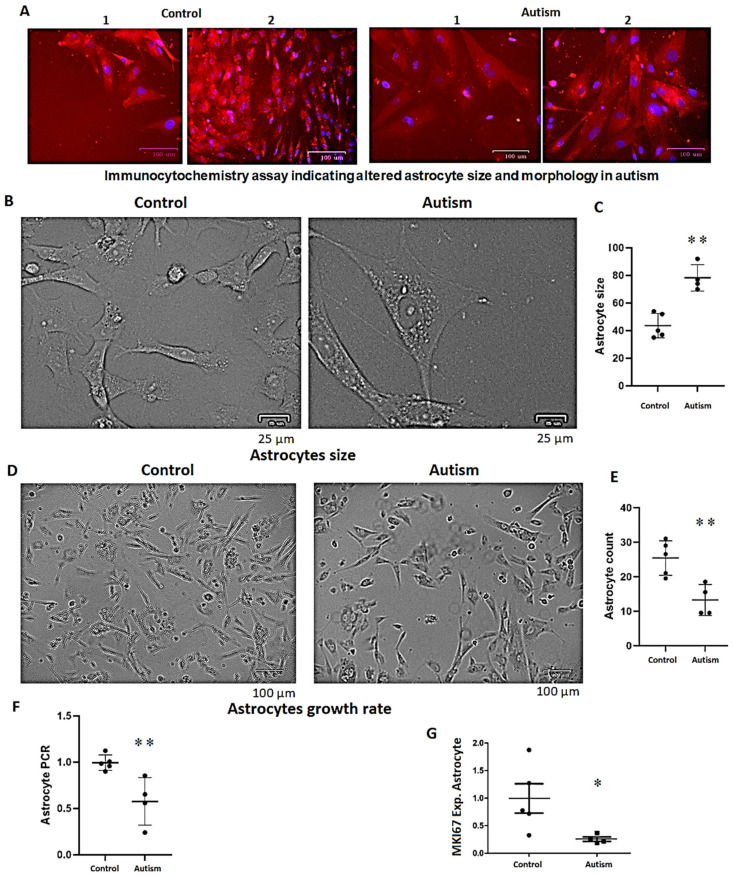
Astrocyte size and growth rate in controls vs. patients with autism. (**A**) Immunocytochemistry assay showing differences in the size of astrocytes in two representative samples of controls and patients with autism (scale bars represent 100 µm both for autism and the control subject). (**B**) shows a larger magnification of astrocytes in culture (scale bars represent 25 µm) and (**C**) shows the average size of astrocytes in autism is larger than control subjects (*p* = 0.0035), as randomly measured for 6 astrocytes per well (in triplicate for each case or control subject, a total of four cases and five controls) using the same microscope magnification. (**D**) illustrates the confluency of astrocytes following a 2-week differentiation period using an equal number of NSCs. Note that here, astrocyte size is also larger in autism. (**E**,**F**) depict the growth rate of astrocytes from control subjects compared to subjects with autism using a cell counting method in triplicate (**E**) or qPCR amplification of extracted DNA normalized to the mean CT value of the control subjects (**F**), as outlined in the Methods section. (**G**) exhibits reduced expression of *MKI67* (a marker for cell proliferation) in autism. *p*-values between 0.05 and 0.01 are marked with *, and *p*-values less than 0.01 are marked with **.

NSCs were cultured and differentiated into astrocytes, and after two weeks, their size was assessed as described in Methods. The astrocytes of patients with autism were larger than those of the control subjects (>1.5-fold, *p* = 0.0035, Figure 6A–C). In contrast to the NSCs mentioned earlier, the growth rate of astrocytes was reduced after differentiation in patients with autism according to the MTS assay in triplicate (23%, *p* = 0.001, 1.0 vs. 0.77, SD 0.07 vs. 0.06 in controls vs. autism, respectively). Considering the larger size of astrocytes in autism and the possible improperness of the MTS assay, the estimation of growth rate using the cell counting method and PCR amplification (as described in the method) showed that the growth rate of astrocytes in patients with autism is nearly half that of the control subjects (Figure 6D–F). *MKI67* gene expression analysis (a marker for cell proliferation) revealed ~75% reduced expression of *MKI67* in autism vs. controls (Figure 6G). In the context of long-term survival, the differentiated astrocytes could barely maintain their growing capacity and survive more than 16 weeks in patients with autism, but the astrocytes of the control subjects could survive more than six months upon multiple passages.

## 4. Discussion

We have identified differences between iPSC-derived NSCs, neurons and astrocytes from subjects with autism vs. controls, which together support the potential value of this model approach. Significant differences include a disorganized colony formation and an increased cell growth rate in the NSCs of subjects with autism, along with reduced expression of *EN2* and higher expression of *BDNF* (a growth-supporting gene), in the NSCs as well as astrocytes of patients with autism compared to the control subjects. Considering *EN2*’s role in pattern formation during the central nervous system development [36], its decreased expression in the NSCs of patients with autism may underlie the disorganized colony formation of NSCs we observed in autism. The higher expression of *BDNF* is consistent with higher expression of this gene reported in multiple studies of autism, including two meta-analyses [8,42], as well as the increased brain size and the number of neuronal cells and their processes observed in the early age of patients with autism [4,5,43]. Nevertheless, in later childhood, their brain weight and cortical thickness are decreased, accompanied by the loss of neuronal arborization and cognitive capabilities [9,10].

Interestingly, we also found a 30-fold decrease in the expression of *NTRK2*, the BDNF receptor gene, in the astrocytes of patients with autism. In this context, a genetic study of 469 trio families reported that genetic polymorphisms of *NTRK2* are linked to autism pathogenesis [44]. Furthermore, a study using laser capture microdissection of postmortem brains reported that *NTRK2* expression was significantly lower in pyramidal neurons of the anterior cingulate cortex of patients with autism but not in the astrocytes of the anterior cingulate cortex and neurons of the frontal cortex of these patients [45]. However, the sample size in this study was small (8 per group), and the astrocytic expression level of *NTRK2* was quite variable both in cases and controls [45]. While our findings suggest that astrocyte’s reduced growth rate may be due to the decreased expression of *NTRK2* in these cells, its reduced expression is likely due to DNA hypermethylation, as shown in the postmortem brains of patients with bipolar disorder [46]. However, more studies are required to confirm this assumption.

We also observed higher expression of *IL6* in NSCs, astrocytes, and neurons in patients with autism. At least one study reported that *IL6* “regulates adult neural stem cell numbers during normal and abnormal post-natal development” and its blockade causes a long-term decrease in forebrain NSCs [47]. Thus, in addition to inflammatory activity, *IL6* contributes to NSC growth, and its higher expression may induce the higher growth rate of NSCs in autism observed in our studies. However, in the differentiated cells, its higher expression, associated with higher inflammatory reactions, may impact the growth rate of the neurons and astrocytes of patients with autism. Notably, higher levels of IL6 were also reported in the brain, blood cells, and plasma of patients with autism [48,49,50].

In contrast to *BDNF* and *IL6*, we observed decreased expression of *IFI16*, an anti-inflammatory gene, in the NSCs of patients with autism. However, after differentiation, *IFI16* exhibited significant up-regulation in the astrocytes, whereas *IL6* was upregulated in both astrocytes and neurons of patients with autism. This was accompanied by a reduced rate of neuronal growth, bifurcation, network formation, and reduced number of spines and likely synapses as *SYN1* expression was reduced by almost 50% in autism. These findings suggest that while increased expression of inflammatory genes may impact astrocyte and neuron health in autism, the increased expression of *IFI16* might be a secondary reaction. Moreover, the astrocytes of patients with autism exhibited higher expression of *TGFB1* and *TGFB2* but reduced expression of proliferator genes such as *CXCR4* and *NURR1* in addition to *RELN*, *EN2*, *HAP1*, *SIRT1*, and *GPX1* vs. controls. The impact of this expression pattern on brain cellular functions is further elaborated below.

Regarding TGFB genes, an increase in protein levels of TGFB1 was reported in postmortem brains and CSF of patients with autism [16]. *TGFB1* is a negative modulator of adult neurogenesis and a long-lasting inhibitor of neural stem and progenitor cell proliferation [51]. *TGFB1* also mediates the induction of the *GFAP* promoter gene (an astrocytic gene) in response to glutamate (upon neuronal activation), which is involved in astrocyte differentiation [52]. Likewise, *TGFB2* has antiproliferative effects on hippocampal granule cells, which typically renew throughout life [53]. It is also known that *TGFB1* inhibits *RELN* expression through snail, which decreases both promoter activity and *RELN* expression [35]. Our data indicate an inverse correlation between the expression of *SNAI1* and *RELN* in control subjects. Nonetheless, there was no expression alteration of *SNAI1* in NSC, astrocytes, or neurons. This suggests that reduced expression of *RELN* in the astrocytes of patients with autism is mediated by TGFB-mediated induction of snail activity. Notably, RELN as an extracellular matrix protein with reduced expression in the brain of patients with autism, is involved in cell positioning, neuronal migration, and dendritic spine formation [29,54,55], processes impacted in our in vitro autism studies, exhibited a highly significant reduced expression along with increased expression of *TGFB1* as well as *TGFB2* in astrocytes. In our studies using primers covering CpGs located between –523 and –260 at the start of the coding region, the *TGFB1* promoter region was unmethylated in astrocytes but partially methylated in neurons. While nearly half of this DNA methylation was due to 5-hmC, an almost 100% increase in 5-hmC level in autism was not statistically significant due to the small sample size and variability in 5-hmC level. Nevertheless, a human study reported DNA hypomethylation of *TGFB1* in the blood cells of patients with autism [56]. Thus, it is likely that the increased expression of *TGFB1* in the astrocytes of patients with autism is mediated by DNA hypomethylation of other CpG sites or/and due to different epigenetic mechanisms. Meanwhile, it is important to note that unmethylation of the *TGFB1* promoter at this site in astrocytes and its methylation in neurons signifies the identity of astrocytes vs. neurons and implies that the methylation signal in postmortem brain samples originates from the neuronal cells rather than astrocytes and that 5-hmC might be a regulatory mechanism to fine-tune *TGFB1* expression in neurons. Notably, 5-hmC is a transitional product during the demethylation of DNA. There are several reports on its abundance in the brain, and it induces gene expression [57,58] even before being converted to unmethylated cytosine [59].

Considering *TGFB2*, similar to *TGFB1*, we identified neuronal-specific DNA methylation signature vs. astrocytes at site B with an increased 5-hmC level in neurons. Additionally, we found DNA hypomethylation of site A of the *TGFB2* promoter region in the postmortem brains of patients with autism and astrocytes of patients with autism associated with its increased expression. Another study also found that promoter DNA hypomethylation of *TGFB2* in the postmortem brains of patients with schizophrenia and bipolar disorder is associated with its increased expression [33]. Hence, more studies on molecular or environmental events that lead to DNA hypomethylation of *TGFB2* promoter may uncover one of the underlying mechanisms of disease pathogenesis in these interlinked psychotic disorders. From a mechanistic point of view in autism, the activity of TGFB signaling genes is tightly linked to *mTOR*, whose increased expression affects *TSC1* (a partner of *HAP1*) linked to autism neuropathology [60]. Our experiments with iPSCs-derived astrocytes and neurons also revealed that in addition to altered expression of *TGFB1&2*, the expression of *HAP1* was significantly reduced (~60%) in autistic astrocytes and neurons vs. controls. Furthermore, *HAP1* promoter DNA methylation was increased in the postmortem brains and neurons of patients with autism vs. controls. In contrast, in astrocytes, its promoter 5-hmC level was decreased (i.e., more 5-hm, which is associated with a decrease in gene expression). These findings suggest that TGFB-mediated *HAP1* gene silencing, possibly via the induction of its promoter DNA methylation in neurons, may play an important functional role in autism pathogenesis, as *HAP1* is a partner of *TSC1* (tuberous sclerosis 1), a gene linked to autism phenotype [60,61].

It has also been reported that TGFβ induces the expression of *SLC1A2* (*GLT1*) in mouse astrocytes and increases astroglial glutamate uptake [62]. However, we found reduced expression of *SLC1A2* in iPSC-derived astrocytes in autism (Table 2), as well as DNA hypermethylation of its promoter in the postmortem brains of patients with autism (Table 3 and Figure 2M). Consistent with our findings, there are some reports that reduced expression of *SLC1A2* contributes to the pathogenesis of autism and other major mental diseases [63] and that the deletion of 11p14-p12, which includes *SLC1A2*, is linked to autism phenotype [64]. In mice, astroglial glutamate transporter deficiency induced by an astrocyte-specific *GLT1* knockout also increases synaptic excitability associated with repetitive behaviors, a phenotype frequently observed in autism [65]. While these studies generally support a link between reduced SLC1A2 activity and autism pathogenesis, the unexpected lack of correlation between the expression of *TGFB* genes and *SLC1A2* in our study suggests that an unrelated epigenetic silencing of *SLC1A2* in autism may disrupt the link between *TGFB* expression and *SLC1A2*. At least in astrocytes, it was shown that *SLC1A2* promoter DNA methylation suppresses its expression [66]. DNA hypermethylation of the *SLC1A2* promoter region was also shown in the blood cells of patients with bipolar disorder. However, patients with nicotine addiction exhibited reduced DNA methylation [67]. In contrast to bipolar disorder, increased expression of *SLC1A2* (along with *TGFB2*) has been linked to schizophrenia pathogenesis [33]. Altogether, these findings warrant further investigations in this era, particularly pertinent to the epigenetic silencing of *SLC1A2*, as its deletion and knockout are linked to autism phenotype in humans and mice [64,65].

We also found compromised survival of neurons and astrocytes derived from patients with autism. In relation to the underlying mediates of the impacted cells’ survivals, *CXCR4* and *SIRT1* (a gene involved in epigenetic regulation) are known to be neuro-protective [68,69]. It is also known that “a nurr1/corest pathway in microglia and astrocytes protects dopaminergic neurons from inflammation-induced death” [70]. Nurr1, in addition to being essential for synapse formation and neuronal migration [71], which were altered in autism in our studies, inhibits the inflammatory effects of NF-*κ*B as well [72]. Interestingly, all three of these genes, along with *SNCA* (alpha synuclein), which mediates inflammatory and immune responses [73], exhibited highly significantly reduced expression in our iPSC-derived astrocytes of patients with autism. SIRT1 also plays a critical role in telomere maintenance during stem cell aging due to its contribution to gene silencing. It thus has a neuro-protective function in disease models of Alzheimer’s disease, Huntington’s disease, and amyotrophic lateral sclerosis [74,75]. In our studies, while the NSC growth rate was higher in patients with autism, *SIRT1* was not affected in NSCs, though it was severely reduced in the astrocytes of patients with autism. This indicates that its dysregulation begins during or after astrocyte differentiation, potentially compromising astrocyte growth rate and survival in autism (Figure 6).

Considering these findings, it is conceivable that in the brain of patients with autism, inadequate astrocyte survival at an early age may result in insufficient neuronal protection and excessive synapse pruning, which involves the inflammatory complement genes, *C1Q* and *C3*, both epigenetically up-regulated in the postmortem brains of patients with autism [76]. This may, in turn, impact neuronal network formation, which has been reported to be affected in patients with autism [54] and observed in our studies, as shown in Figure 3 and Figure 4. However, it is essential to acknowledge that, due to the intricate branching patterns of neurons, one of the primary limitations of our studies is the absence of quantification regarding the number of synapses per neuron. Consequently, despite the reduced expression of *SYN1* in autism, this could be attributed to the diminished neuronal arborizations associated with autism. Similarly, the observed smaller spine size may stem from the delayed neuronal growth rate in autism, emphasizing the need for more cautious interpretations of these findings.

Regarding *IFI16*, an anti-inflammatory gene, we found a highly significantly reduced expression in the NSCs (75%) but a significantly increased expression in the astrocytes (>45%) of patients with autism vs. controls. This is in line with other studies reporting increased levels of IFI16 in plasma and its hyper-expression in the brain of patients with autism patients [25,26]. Based on our data, astrocytes express *IFI16* almost 7-fold more than neurons. Therefore, astrocytes might be the origin of an overall higher *IFI16* expression in the brains of patients with autism. *IL4*, another anti-inflammatory gene, exhibited deficient expression levels in our study cell lines and an almost 80% decreased expression in the astrocytes of patients with autism vs. controls. It has been shown that *IL4* expression is increased in the blood cells of mothers during pregnancy whose children develop autistic symptoms at age 7 years [77]. *IL4* was also higher in newborns with low birth weight who developed an autistic phenotype in later life [78]. While increased expression of this anti-inflammatory gene in pregnant mothers or newborns could be the response of white blood cells to an ongoing inflammatory reaction, its reduced expression in our iPSC-derived neurons or astrocytes may indicate an intrinsic deficit in brain cells, and astrocytes in particular, for *IL4* expression in patients with autism, which makes their brain cells vulnerable to inflammatory damages. This may be intensified by reduced expression of *GPX1*, which acts against inflammation-induced oxidative stress [38]. Indeed, our gene expression and epigenetic findings, coupled with the larger sizes of iPSC-derived astrocytes from individuals with autism, represent activated astrocytes. This observation aligns with reports from other studies conducted on postmortem brain samples [16]. Nevertheless, further studies with larger sample sizes, accompanied by cell karyotyping and the implementation of different quality control measures, particularly for the iPSC lines (an aspect among the limitations of this study), are necessary to confirm whether iPSC-derived differentiated cells are appropriate surrogates for studying disease pathogenesis or developing patient-specific therapeutics in autism.

## 5. Conclusions

We found more extensive examples of differential gene expression and epigenetic alteration in the iPSC-derived astrocytes of patients with autism than in NSCs and neurons. These alterations were heavily skewed toward inflammatory processes and were associated with larger cell size but reduced viability of astrocytes. Our findings suggest that iPSC-derived astrocytes from patients with autism reflect activated astrocytes. Our data also indicate that epigenetic alterations may play a significant role in the dysregulation of key inflammatory genes, such as *IL4*, *IL6* and *TGFB2*. Colony formation and cell–cell signaling were also compromised in NSCs and neurons from patients with autism, along with expression and/or epigenetic alterations of some critical genes such as *RELN* reported in clinical samples from these patients and/or in our limited postmortem brain samples. Hence, it can be concluded that iPSC-derived NSCs, neurons and astrocytes may fairly represent molecular alterations observed in autism, consistent with a transcriptomic study of the iPSC-derived neurons of patients with autism [79]. Upon confirmation in other studies with larger sample sizes, patient-specific iPSC-derived brain cells can be used to identify diagnostic and therapeutic biomarkers for this syndromic neuro-developmental disorder with diverse etiopathologies.

## Figures and Tables

**Table 1:** Information of the controls and patients who donated samples for iPSC generation.

Control				Autism			
ID	Age/Year	Sex	Sources	ID	Age/Year	Sex	Sources
SV0000525	6	Female	Simon’s foundation	SV0000540	14	Male	Simon’s foundation
SS0013111	45	Male	Simon’s foundation	SS0013099	14	Male	Simon’s foundation
SV0001455	12	Male	Simon’s foundation	SV0001473	6	Female	Simon’s foundation
SV0001464	3	Female	Simon’s foundation	SC171	Unknown	Male ×	NIMH
SC173	Unknown	Male **×**	NIMH	SC126	Fragile ×	Male ×	NIMH

**×** SRY gene expression analysis was used to determine the sex of these samples.

**Table 2:** Expression changes in iPSC-derived astrocytes and neurons in patients with autism (4 lines) vs. control subjects (5 lines).

	Astrocyte	Neuron	Expression Ratio
Gene	Fold change in autism (Mean ± SEM of controls/cases, 95% CI) t, ***p*** value	Fold change in autism (Mean ± SEM of controls/cases, 95% CI) t, ***p*** value	Astrocyte/neuron ratio in controls
***TGFB1***	2.3 (0.94 ± 0.17/2.3 ± 0.34, −2.2 to −0.54) t = 3.9, ***p* = 0.006**	1.5, not significant (NS)	6
***TGFB2***	2 (1 ± 0.17/2 ± 0.4, −2.197 to −0.3160) t = 3.2, ***p* = 0.02**	1.4 (NS)	>3
***TGFB3***	NS	NS	2.2
***NR2E1***	0.36 (1 ± 0.3/0.37 ± 0.07, −0.13 to 1.4) ***p* = 0.09**	NS	>15
***IL4***	0.21 (1.06 ± 0.31/0.17 ± 0.06, 0.05 to 1.7) t = 2.5, ***p* = 0.04**	NS	3.5
***IL6***	34 (1.1 ± 0.3/37 ± 7.4, −51 to −20) t = 5.5, ***p* = 0.001**	28 (1 ± 0.16/28 ± 0.2.8) ***p* = 0.053**	>30
***IL1B***	NS	NS	2
***TNFA***	NS	No expression	N/A
***TNFRSF1A***	NS	NS	>1.5
***IFI16***	1.5 (0.99 ± 0.12/1.5 ± 0.14, −0.97 to −0.12) t = 3, ***p* = 0.02**	NS	~7
***IGFBPL1***	0.04 (1.1 ± 0.38/0.03 ± 0.01, −0.08 to 2) t = 2.5, ***p* = 0.04**	NS	>2
***GPX1***	0.6 (1 ± 0.1/0.6 ± 0.1) t = 3.2, ***p* = 0.014**	0.7 (1.1 ± 0.11/0.78 ± 0.13, −0.08 to 0.7) t = 1.9, ***p* = 0.1**	1
***SIRT1***	0.32 (1.05 ± 0.1/0.32 ± 0.06, 0.44 to 1) t = 5.8, ***p* = 0.0006**	NS	1
***NURR1***	0.1 (1 ± 0.3/0.07 ± 0.02, 0.25 to 1.6) t = 3.2, *p* **= 0.01**	NS	2
***SNCA***	0.4 (1 ± 0.1/0.4 ± 0.1, 0.27 to 0.94) t = 4.4, ***p* = 0.004**	NS	1.5
*BDNF*	1.8 (0.94 ± 0.2/1.8 ± 0.15, −1.5 to −0.26) t = 3.4, ***p* = 0.01**	NS	3
*CXCR4*	0.2 (1 ± 0.3/0.18 ± 0.1, 0.3 to 1.3) t = 3.9, ***p* = 0.006**	NS	3
*EN2*	0.2 (1 ± 0.25/0.24 ± 0.19, −0.01 to 1.5) t = 2.23, ***p* = 0.052**	3.3 (1 ± 0.3/3.6 ± 1.2, −5.2 to −0.002) t = 2.4, ***p* = 0.05**	>20
*HAP1*	0.25 (1 ± 0.08/0.25 ± 0.1, 0.52 to 1.1) t = 6.3, ***p* = 0.0004**	0.4 (1 ± 0.26/0.4 ± 0.14, −0.18 to 1.3) t = 1.8, ***p* = 0.11**	0.5
*RELN*	0.032 (1 ± 0.2/0.035 ± 0.02, 0.1 ± 0.16) t= 5.9, ***p* = 0.0006**	NS	0.5
*NTRK2*	0.04 (1 ± 0.2/0.04 ± 0.01, 0.35 to 1.5) t = 3.8, ***p* = 0.01**	NS	2.9
*SLC1A2*	0.11 (1.03 ± 0.35/0.1 ± 0.08, 0.07 to 1.75) t = 2.6, ***p* = 0.045**	0.5 (1 ± 0.24/0.5 ± 0.14, −0.27 to 1.141) t = 1.5, ***p* = 0.18**	2
*SLC1A3*	0.11 (1 ± 0.15/0.11 ± 0.04, 0.45 to 1.3) t = 4.9, ***p* = 0.002**	NS	>4
*SNAI1/Snail*	NS	NS	1.5
*SYN1* (synapsin)	N/A	0.52 (1 ± 0.17/0.5 ± 0.17, −0.1 to 1) t = 1.9, ***p* = 0.1**	0.045

Note: The first 15 genes (in bold) are linked to inflammatory reactions.

**Table 3:** Total DNA methylation (5-mc) and 5-hydroxymethylation (5-hmc) changes in iPSC-derived astrocytes and neurons in autism (4 lines) vs. controls (5 lines).

	Astrocytes		Neurons	
Gene	5-mc (Mean ± SEM Cont./Autism, 95% CI) t, ***p*** value	5-hmc (Mean ± SEM Cont./Autism, 95% CI) t, ***p*** value	5-mc (Mean ± SEM Cont./Autism, 95% CI) t, ***p*** value	5-hmc (Mean ± SEM Cont./Autism, 95% CI) t, ***p*** value
*TGFB1*	Unmethylated	Unmethylated	NS (no significant change)	NS
*TGFB2* Site A	Hypomethylation (1 ± 0.1/0.58 ± 0.13, 0.1 to 0.7) t = 3.2, ***p* = 0.04**	Negligible 5-hmc	NS	NS
*TGFB2* Site B	Unmethylated	Unmethylated	Heavily methylated, 50% more in autism but NS	~50% more in autism (1 ± 0.1/1.460 ± 0.11, 0.8 to −0.08), t = 2.9, ***p* = 0.03**
*IL6*	Hypomethylated (0.95 ± 0.17/0.46 ± 0.03, 0.02 to 1), t = 2.5, ***p* = 0.04**	NS	NS	NS
*TNFA*	Hypomethylated (1 ± 0.2/0.34 ± 0.03, 0.07 to 1.2), t = 2.6, ***p* = 0.03**	NS	NS	NS
*IFI16*	No methylation	No 5-hmc	Hypomethylated (1 ± 0.14/0.48 ± 0.11, 0.08 to 0.96) t = 2.8, ***p* = 0.02**	NS
*EN2*	Hypomethylated (1 ± 0.15/0.53 ± 0.025, 0.09 to 0.86) t = 2.9, ***p* = 0.02**	NS	NS	NS
*HAP1*	NS	Decreased (1 ± 0.15/0.5 ± 0.05, 0.1 to 0.96) t = 2.9, ***p* = 0.02**	Hypermethylated (1 ± 0.3/2.5 ± 0.5, −2.7 to −0.2) t= 2.7, ***p* = 0.04**	NS
*RELN*	10% methylation, NS	Negligible 5-hmc	NS	Negligible 5-hmc
*SLC1A2*	Negligible methylation	Negligible 5-hmc	NS	Negligible 5-hmc
*SLC1A3*	No methylation	No 5-hmc	Hypomethylated (1 ± 0.1/0.6 ± 0.11, 0.04 to 0.75) t = 2.6, ***p* = 0.035**	NS

## Data Availability

Available upon request to interested researchers.

## Supplementary Materials

The following supporting information can be downloaded at: https://www.mdpi.com/article/10.3390/cells13131095/s1, Table S1: Primers that were used for expression analysis. Table S2: Primers for 5-mc and 5-hmc analysis, designed from the promoter regions of candidate genes. Figure S1: Correlation analysis for GAPDH and Actin expression levels. Figure S2: Enlarged view of dendritic arborizations and spines (representative example) for measuring spine size and density.
