# Supplementary material for: iPSC-Derived Astrocytes and Neurons Replicate Brain Gene Expression, Epigenetic, Cell Morphology and Connectivity Alterations Found in Autism
Source: Cells. 2024 Jun 25;13(13):1095. doi: 10.3390/cells13131095 (PMC11240613; doi:10.3390/cells13131095)
Supplement: Supplementary file 1 [file cells-13-01095-s001.zip › Supplementary Tables S1 and S2.pdf]

Supp. Table S1. Primers used for gene expression analysis			
Gene	Forward primer	Reverse primer	Amplicon size
TGFB1	CTAATGGTGGAAACCCACAACG	TATCGCCAGGAATTGTTGCTG	209
TGFB2	AGAGTGCCTGAACAACGGATT	CCATTCGCCTTCTGCTCTT	116
TGFB3	GGAAAACACCGAGTCGGAATAC	GCGGAAAACCTTGGAGGTAAT	121
NR2E1	CAAACGGAGCATCCGAAGGAA	GTCCACGGAAGTAGAGGGC	209
GPX	CAGTCGGTGTATGCCTTCTCG	GAGGGACGCCACATTCTCG	105
IL4	CGGCAACTTTGTCCACGGA	TCTGTTACGGTCAACTCGGTG	111
IL6	ACTCACCTCTTCAGAACGAATTG	CCATCTTTGGAAGGTTCAGGTTG	149
IGFBPL1	TCGTTCCTCCCCGAAGTGTT	GACAGCTATATTGACATGGTCCC	167
IL1B	AGCTACGAATCTCCGACCAC	CGTTATCCCATGTGTCGAAGAA	186
TNFA	GAGGCCAAGCCCTGGTATG	CGGGCCGATTGATCTCAGC	91
TNFRSF 1A	TCACCGCTTCAGAAAACCACC	GGTCCACTGTGCAAGAAGAGA	96
IFI16	TAGAAGTGCCAGCGTAACTCC	TGATTGTGGTCAGTCGTCCAT	179
BDNF	CTACGAGACCAAGTGCAATCC	AATCGCCAGCCAATTCTCTTT	147
NTRK2	ACCCGAAACAAACTGACGAGT	AGCATGTAAATGGATTGCCCA	91
EN2	CCGGCGTGGGTCTACTGTA	CCTCTTTGTTCGGGTTCTTCTT	91
HAP1	TGAGCGCGCCGCCTTTATTCG	TGACGTCTTCCTGGGTGATCTTTT	89
NURR1 (NR4A2)	GCACTCCGGGTCGGTTTAC	GCCACGTAGTTCTGGTGGAA	129
RELN	GCGAGGTGCTCATTTCCCTGCATATT	GTGTATAGTCCTGTCACCAGCAAGC	124
SLC1A2	CCTTGTCCAAGCCTGCTTTCA	CTCAGTCACAGTCTCGTTCAAC	127
SLC1A3	AGCAGGGAGTCCGTAAACG	AGCATTCCGAAACAGGTAACTTT	91
SNCA	GACAAAAGAGGGTGTTCTCTATGTAG	GCTCCTCCAACATTTGTCACTT	111
SYN1	AGTTCTTCGGAATGGGGTGAA	GTCCCCAGTTTCTTATGCAGTC	175
SIRT1	TGCGGGAATCCAAAGGATAATTCAGTGTC	CTTCATCTTTGTCATACTTCATGGCTCTATG	236
Survivin (BIRC5)	GCCCAGTGTTTCTTCTGCTT	TCTCCGCAGTTTCCTCAAAT	140
SRY	GCGTATTCAACAGCGATGATTAC	TCTCCCGTTTCACACTGATACTT	121
GFAP	AGGTCCATGTGGAGCTTGAC	GCCATTGCCTCATACTGCGT	82
SOX2	GCCGAGTGGAAACTTTTGTCG	GGCAGCGTGTACTTATCCTTCT	155
MAP2	CGAAGCGCCAATGGATTCC	TGAACTATCCTTGCAGACACCT	161
DCX	TCCCGGATGAATGGGTTGC	GCGTACACAATCCCCTTGAAGTA	152
MKI67	GCCTGCTCGACCCTACAGA	GCTTGTCAACTGCGGTTGC	127
β-Actin	CGAGCACAGAGCCTCGCCTTTGCC	TGTCGACGACGAGCGCGGCGATAT	94
GAPDH	ACAACTTTGGTATCGTGGAAGG	GCCATCACGCCACAGTTTC	101
Note: The sequence of primers taken from the Harvard primer bank with the exception of β-actin, BIRC5, TGFB2, HAP1 and RELN			

Supp. Table S2. Primers for 5-mc and 5-hmc analysis designed from the promoter regions of the candidate genes			
Gene	Forward primer	Reverse primer	Amplicons size
TGFB1	TTCCATCCTTCAGGTGTCCTGTTG	GGTGTGGGTCACCAGAGAAAGAG	205
TGFB2 Site A	CCTTTTACCATGAAGACTGTAGAGAC	TCAAATGATCAGTTCTTTGAAGACCT	170
TGFB2 site B	CGTGGTTCAGAGAGAACTTATAAATCTCC	TCTGTCTTTCTCTTGTCAGGAGC	119
EN2	AACGGGGTTCCCGGGTCAGT	GAACGACCGCCGCCCTCAAG	109
RELN	TTTGACGTCCCTCGCAGAAGAGT	TGGCTCGGCGGCACCT	165
HAP1	CGTCACCACTAGCTCCCCTCCC	GAGTCTGCCGTCCGCTGC	144
TNFA	AAGACTGAAACCAGCATTATGAG	GGACAAGCCTGGGACAGC	118
IFI16	CTCCTATTATAAAGTTTGCTTTTTTGGC	ACAGGCACACATGACCATAC	187
IL6	AGGTGGGTAGGCTTGGC	CGTTGGCCTCAAATCTACAGG	134
CXCR4	CACCTGTCTTCAGGCGCATC	GGACCCTGCTGTTTGCGGGT	106
SLC1A2 site A	GGTGAGTGTGAGCTGAAGC	CTCTCGCCATAAATTAGCCAAATAAGA	262
SLC1A2 site B	CGTTGAGGCGCTAAAGGGCTT	CTCACCCTTCCGTAGATGCCAT	186
