# Supplementary figures and images for: iPSC-Derived Astrocytes and Neurons Replicate Brain Gene Expression, Epigenetic, Cell Morphology and Connectivity Alterations Found in Autism
Source: Cells. 2024 Jun 25;13(13):1095. doi: 10.3390/cells13131095 (PMC11240613; doi:10.3390/cells13131095)

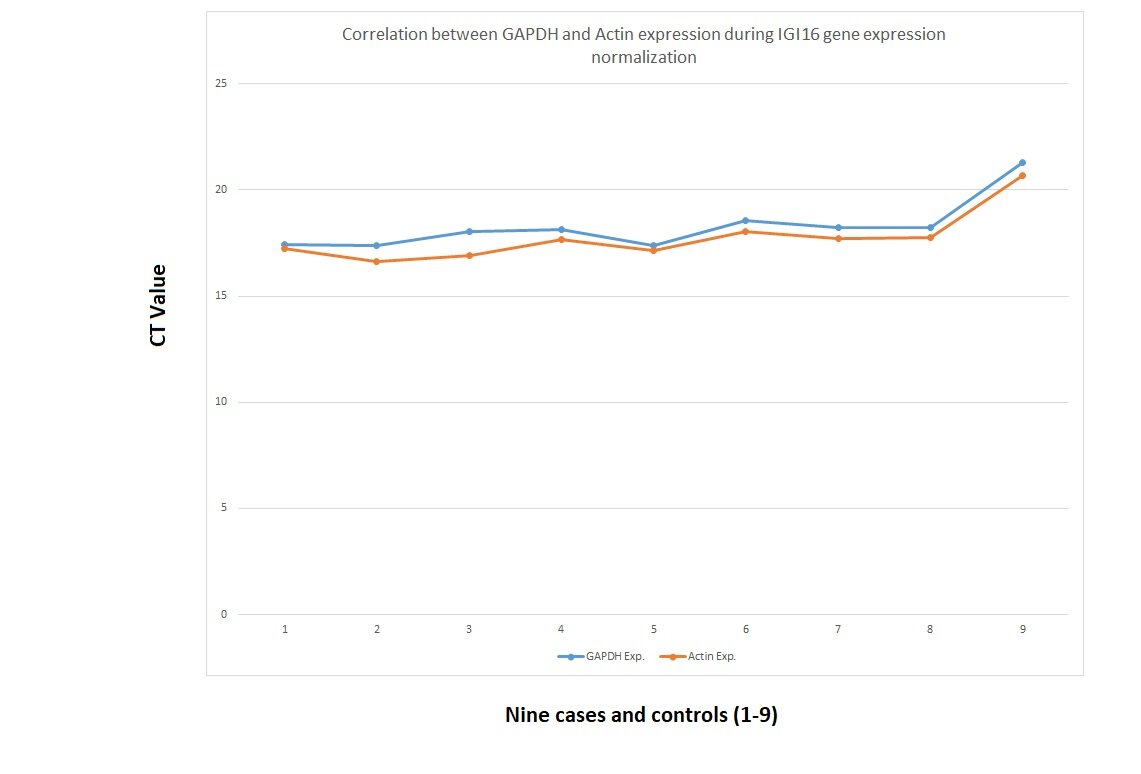

Supplement: Supplementary file 1 [file cells-13-01095-s001.zip › Supp Fig S1.jpg]

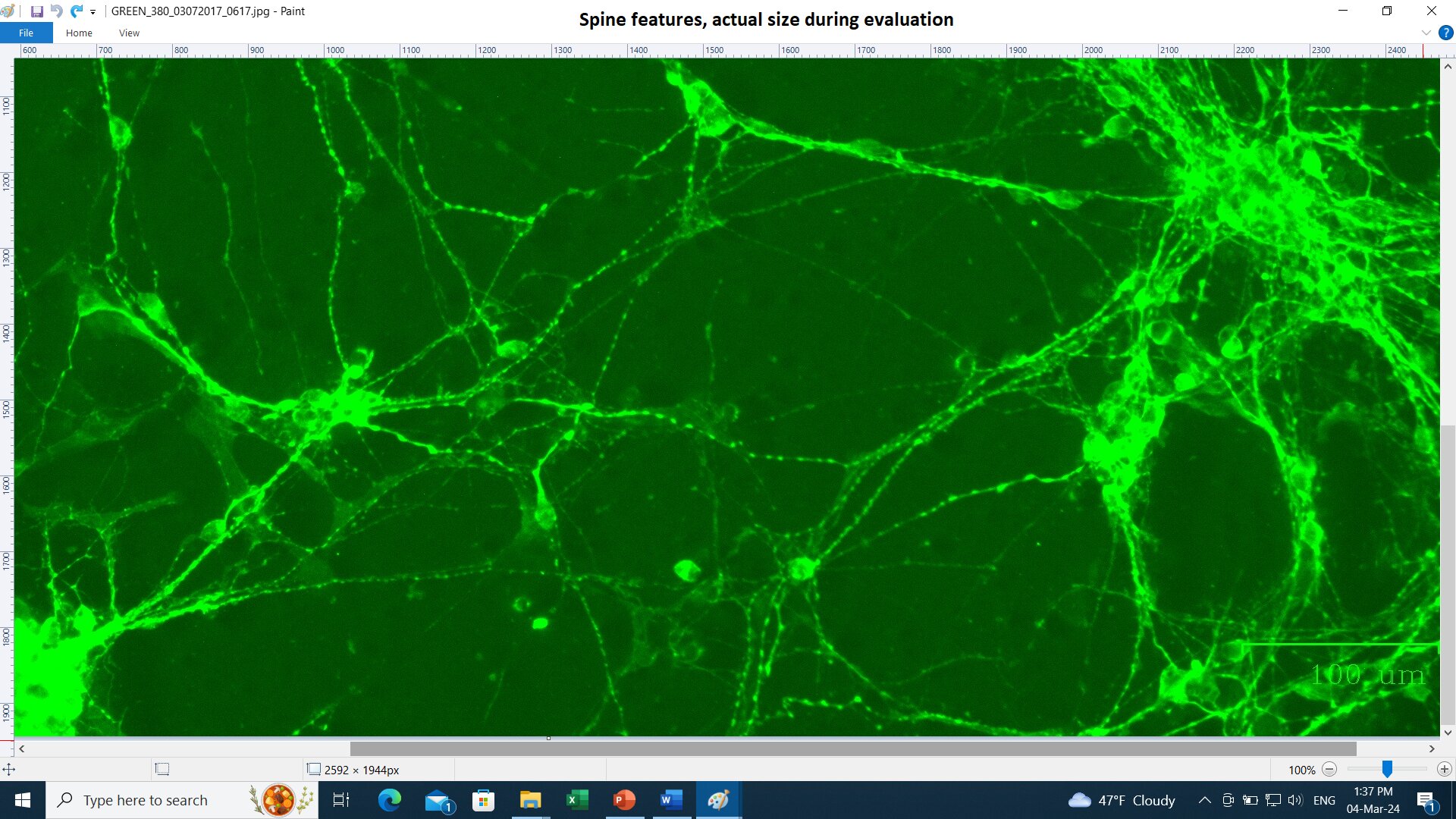

Supplement: Supplementary file 1 [file cells-13-01095-s001.zip › Supp Fig S2-spines.jpg]
